# Surgical treatment for arachnoid cysts in adults: Clinical, radiological, neurocognitive and patient-reported outcomes

**DOI:** 10.1016/j.bas.2026.106077

**Published:** 2026-05-01

**Authors:** Lea Pacan, Tim Jonas Hallenberger, Raphael Guzman, Florian Ebel, Jehuda Soleman

**Affiliations:** a Faculty of Medicine, University of Basel, Basel, Switzerland; b Department of Neurosurgery, University Hospital of Basel, Basel, Switzerland; c Department of Pediatric Neurosurgery, University Children's Hospital of Basel, Basel, Switzerland

**Keywords:** Intracranial arachnoid cyst, Neurocognition, Neuropsychology, Patient-reported outcome measures, Neurosurgery, Case series

## Abstract

**Introduction:**

Intracranial arachnoid cysts (AC) are usually incidental findings, most often managed conservatively. Evidence of the effectiveness of surgical management in symptomatic patients is limited.

**Research question:**

This case series evaluates clinical, radiological, neurocognitive, and patient-reported outcomes (PROMs) of surgically treated AC patients.

**Methods:**

Retrospective case series of surgically treated adult AC patients between 2010 and 2023. The primary outcome was improvement of presenting symptoms. Secondary outcomes included cyst volume, neurocognitive outcomes, revision rates, PROMs, and morbidity.

**Discussion:**

Between 2010 and 2023, 2537 AC were radiologically diagnosed at the University Hospital Basel, 18 patients (0.71%) underwent surgical treatment. Mean age was 51 ± 16 years. Cyst locations included convexity (44.4%), retrocerebellar (22.2%), middle fossa (16.7%), cerebellopontine angle (5.6%), and quadrigeminal cistern (5.6%). Common presenting symptoms were headache (66.7%), cognitive deficits (33.3%) and nausea (33.3%). Surgical techniques included endoscopic fenestration (77.8%), microsurgical fenestration (16.7%), and cystoperitoneal shunting (5.6%). During a mean follow-up of 2.9 ± 2.4 years, mean cyst volume decreased by 44.5 ± 30.4%. Clinical improvement was observed in 17 (94.4%) patients. Four (22.2%) patients underwent neuropsychological examinations pre- and post-surgery, showing neurocognitive deficits in the functional domains of memory (100%), concentration (75%), and executive function (50%), all of which improved postoperatively. Three (16.7%) patients required revision surgery due to ventriculitis, hydrocephalus, or insufficient fenestration. Patient satisfaction was high, with 88.9% of patients recommending surgery. No mortality occurred.

**Conclusion:**

Surgical treatment of well-selected AC patients appears to be effective, resulting in improvements in symptoms and neurocognition, with low morbidity rates.

## Highlights

• Surgical treatment of AC improves clinical symptoms in well-selected patients.
• Neuropsychological testing may reveal AC-related deficits missed clinically.
• Neurocognitive and neuropsychological function may improve after surgery.
• PROMs show subjective benefit from surgery and a high patient satisfaction.

## 1 Introduction

Intracranial arachnoid cysts (AC) are fluid-filled malformations of the arachnoid membrane, with a prevalence of 1.4-2.3% in population-based studies (Al-Holou et al., 2013; Weber and Knopf, 2006; Rabiei et al., 2017). Most are asymptomatic and incidentally discovered on cranial imaging (Weber and Knopf, 2006; Kandenwein et al., 2004; Spansdahl and Solheim, 2007; Chen et al., 2016). However, larger cysts or those in eloquent locations may cause symptoms such as headache, seizures, vertigo, nausea, or neurocognitive impairment, mainly due to mass effect or altered cerebrospinal fluid (CSF) dynamics (Al-Holou et al., 2013; Rabiei et al., 2017; Kandenwein et al., 2004; Chen et al., 2016; Pradilla and Jallo, 2007; Schmutzer-Sondergeld et al., 2024; Kershenovich et al., 2018; Torgersen et al., 2010). Management is typically conservative, and surgical treatment is reserved for patients with persistent or progressive symptoms once other etiologies have been excluded (Kandenwein et al., 2004; Chen et al., 2016; Schmutzer-Sondergeld et al., 2024; Cincu et al., 2007; Di Rocco, 2010). Despite the availability of various surgical techniques, including endoscopic or microsurgical fenestration, cystoperitoneal shunt insertion, or stereotactically implanted cysto-ventricular catheters, there is no clear consensus regarding surgical indications or the optimal approach (Spansdahl and Solheim, 2007; Chen et al., 2016; Cincu et al., 2007; Jaradat et al., 2024; Hayes et al., 2019; Kakodkar et al., 2024; Lackermair et al., 2024; Magnéli et al., 2021). This uncertainty is compounded by limited data, particularly with respect to long-term functional and cognitive outcomes.

Further, existing studies rarely assess patient-reported outcomes (PROMs), and to date none have evaluated the effect of surgery on domains such as subjective cognitive functions, sleep, occupational reintegration, or patient satisfaction. A thorough understanding of these factors is essential for a comprehensive understanding of surgical benefit in a typically young, working-age population. The lack of PROM-based data has been increasingly recognized as a critical limitation in the current AC literature (Schmutzer-Sondergeld et al., 2024).

The aim of this study is to present a case series of surgically treated adults with AC, integrating clinical, radiological, neurocognitive, and patient-reported outcomes to provide a multidimensional evaluation of treatment effectiveness.

## 2 Methods

### 2.1 Patient selection

**Fig. 1:**
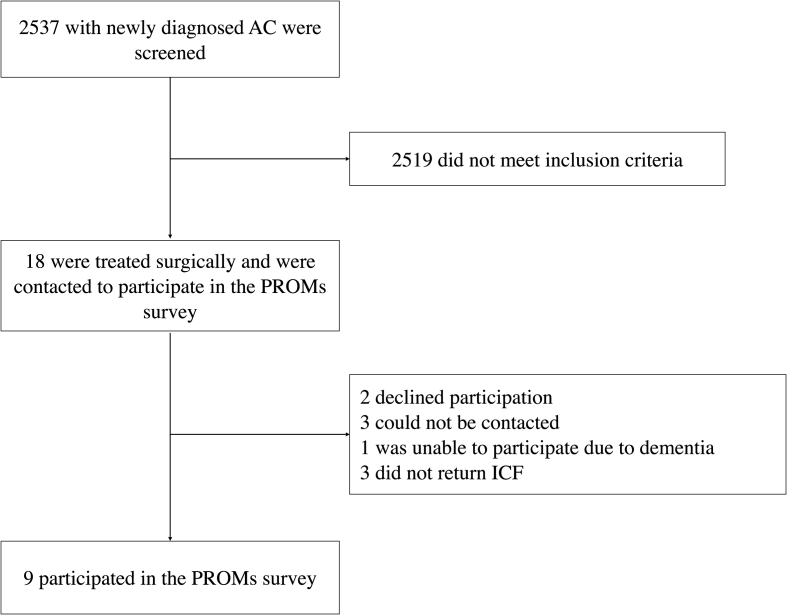
Participant Flow Of 2537 patients with newly diagnosed arachnoid cysts (AC), 2519 did not meet the inclusion criteria. Eighteen patients underwent surgical treatment and were invited to participate in the patient-reported outcome measures (PROMs) survey. Of these, 2 declined participation, 3 could not be contacted, 1 was unable to participate due to dementia, and 3 did not return informed consent. Ultimately, 9 patients were included in the PROMs survey.

Following approval by the local ethics committee (EKNZ, 2024-00545), we conducted a retrospective analysis of all adult patients (≥18 years) who underwent elective surgical treatment for an intracranial AC at the Department of Neurosurgery, University Hospital of Basel, Switzerland, between January 2010 and December 2023 (Fig. 1). This study was conducted in accordance with the PROCESS guideline (Revised Preferred Reporting of Case, 2025).

### 2.2 Primary and secondary endpoints

The primary endpoint was the clinical evolution of the main presenting symptoms and the functional outcome measured by the modified Rankin Scale (mRS), assessed at the last follow-up (mean 2.9 ± 2.4 years). Symptom evolution was categorized as improved, unchanged, or worsened.

Secondary endpoints included surgery-related complications, revision surgery rates, as well as radiological, neurocognitive, and patient-reported outcomes. Radiological outcome was assessed by measuring volumetric changes in cyst size on early postoperative imaging (performed routinely within 24 h of surgery) and at last follow-up compared to the preoperative baseline, using a semi-automated segmentation tool (Sectra Workstation IDS7, Sectra, Linköping, Sweden). For all volume measurements, the CT or MRI sequence with the smallest available slice thickness was selected to ensure maximal segmentation accuracy. Neurocognitive outcomes were evaluated by using standard cognitive screening instruments, such as the Montreal Cognitive Assessment (MoCA) or the Mini-Mental State Examination (MMSE), or through comprehensive neuropsychological assessments conducted by trained neuropsychologists, which assessed core cognitive domains including memory, attention, verbal fluency, visuospatial and executive function. Patient-reported outcomes were collected by L.P. through a structured telephone assessment based on an 84-item, multidimensional questionnaire (see Supplementary Material, PROM questionnaire version 1.2) assessing health-related quality of life, employment status, subjective cognitive function (including memory, arithmetic skills, executive function, emotional regulation, and attention), sleep, pain, and functional independence. During the telephone assessment, patients were asked to rate their condition as better, unchanged, or worse compared to the pre-surgical state. Further patient satisfaction with the surgical outcome, including the willingness to recommend the intervention to others, was assessed.

### 2.3 Indication and procedure

Patients with symptomatic intracranial AC were considered eligible for surgical treatment following a comprehensive clinical evaluation. Surgery was indicated in cases with persistent or progressive symptoms after alternative causes had been excluded, and conservative management was deemed insufficient. Furthermore, the patient's clinical presentation was correlated with changes in AC morphology and volume on serial imaging. Surgical indication due to AC growth was not based on a predefined quantitative threshold but rather on the clinical relevance of volumetric or morphological changes, including increasing mass effect on adjacent brain parenchyma or ventricular structures.

At our institution, intracranial ACs are primarily treated by endoscopic fenestration of the cyst into the cerebrospinal fluid (CSF) compartments, including the ventricular system or subarachnoid cisterns. All procedures were performed under general anaesthesia with the patient positioned supine and the head fixated in a skull clamp (MAYFIELD, INTEGRA LifeSciences Corporation). The location of the cyst is approached by a burr hole followed by the insertion of a peel-away sheath into the cyst cavity under neuronavigational guidance (Brainlab AG, München, Germany). The LOTTA (KARL STORZ SE & Co. KG, Tuttlingen, Germany) or MINOP endoscope (B. Braun SE, Melsungen, Germany) was used. The cyst wall is fenestrated into the selected CSF compartment using either an endoscopic monopolar or scissors. The fenestration is subsequently enlarged with crocodile forceps, a Neuroballoon, or a Fogarty balloon catheter. Intraoperative assessment includes evaluation of the fenestration's pulsation and the pressure equalization between the cyst and surrounding compartments. At the discretion of the surgeon, a ventricular catheter connected to a rickham reservoir and prepared with additional holes along its length may be inserted, with the aim of draining both the ventricle and the cyst. Upon completion of the procedure, the endoscope is removed, and the wound is closed in a standard multilayer fashion. Alternative surgical techniques, including microsurgical fenestration or cystoperitoneal (CP) shunt placement, were employed in cases where endoscopic access was not feasible or in revision surgeries following cyst recurrence or insufficient drainage.

### 2.4 Statistical analysis

All statistical analyses were conducted using IBM SPSS (Version 29.0.2.0). Primary and secondary endpoints were analyzed descriptively. Continuous variables are reported as median with interquartile range (IQR) or mean with standard deviation (±SD), as appropriate. Categorical variables are presented as absolute frequencies and percentages. Group comparisons for continuous variables were performed using the Mann–Whitney *U* test or Kruskal–Wallis test, as appropriate. Categorical variables were compared using the Chi-square test or Fisher's exact test. A p-value <0.05 was considered statistically significant.

## 3 Results

### 3.1 Baseline characteristics

Between January 2010 and December 2023, 2537 adult patients were diagnosed with intracranial ACs on cranial imaging at our institution. Of these, 18 patients (0.71%) underwent surgical treatment for symptomatic ACs, with a mean age of 51 ± 16 years (Fig. 1). Thirteen patients (72.2%) were female. The mean interval between initial diagnosis and surgery was 16.6 ± 28.5 months.

**Fig. 2:**
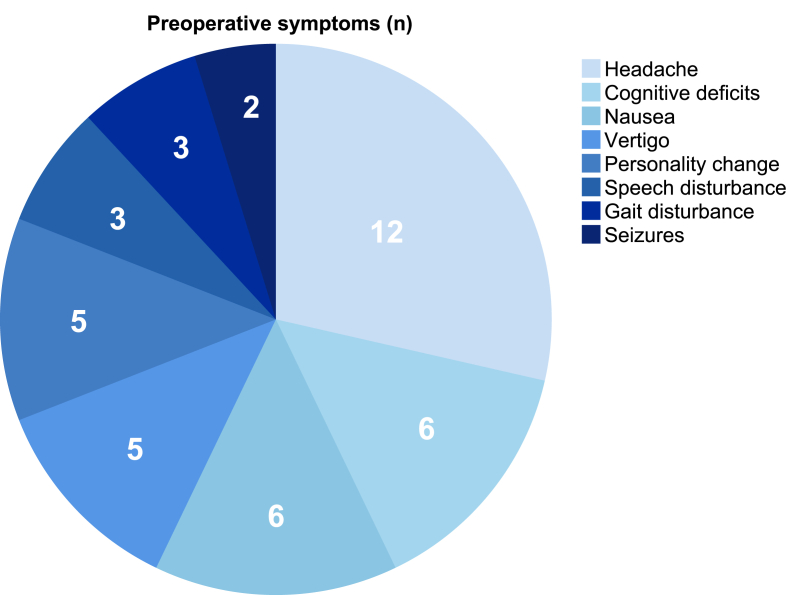
Preoperative symptoms of participants at baseline Distribution of clinical symptoms before surgery in adult patients with arachnoid cysts. Headache was the most frequent symptom (n = 12), followed by cognitive deficits (n = 6), nausea (n = 6), vertigo (n = 5), personality change (n = 5), speech disturbance (n = 3), gait disturbance (n = 3), and seizures (n = 2).

The median preoperative mRS score was 2 (1-3). The most common preoperative symptoms were headache (66.7%), cognitive deficits (33.3%) and nausea (33.3%, Fig. 2). Further symptoms included vertigo (27.8%), personality change (27.8%) and speech disturbance (27.8%). Patients often presented with more than one concurrent symptom. Of the 6 patients reporting cognitive deficits, 4 (22.2%) underwent formal neuropsychological assessment. All demonstrated objective deficits, predominantly in memory (100%), attention and concentration (75%), and executive function (50%). All impairments were classified as mild and did not affect functional dependency in daily life (Table 1). Surgical indication was multifactorial and included persistent (n = 12, 66.7%) or progressive symptoms (n = 10, 55.6%), patient preference (n = 7, 38.9%), and documented cyst growth on serial imaging (n = 6, 33.3%), where volume gain showed clinical correlation.

**Table 1:** Baseline characteristics of the study participants.

	Patients (n = 18)
Age at surgery (median years (range))	54 (20-82)
Sex n (female %)	13 (72.2)

**Cyst location** n (%)	
**Supratentorial AC**	12 (66.7)
convexiy	8 (44.4)
middle fossa	3 (16.7)
bifrontal	1 (5.6)
**Infratentorial AC**	6 (33.3)
retrocerebellar	4 (22.2)
cerebellopontine angle	1 (5.6)
quadrigeminal cistern	1 (5.6)

**Cyst volume** (median cm^3^ (range))	
Overall	36.6 (5.68-184)
Infratentorial AC	69.9 (8.14-184)
Supratentorial AC	20.7 (5.68-33.1)

**Operation type** n (%)	
endoscopic fenestration	14 (77.8)
microsurgical fenestration	3 (16.7)
cystoperitoneal shunt	1 (5.6)

**Clinical presentation**	
Symptom n (%)	
headache	12 (66.7)
cognitive deficits	6 (33.3)
nausea	6 (33.3)
vertigo	5 (27.8)
personality change	5 (27.8)
speech disturbance	5(27.8)
gait disturbance	3 (6.17)

**Pre-operative mRS** (median (range))	2 (1-3)
mRS grouped n (%)	
good (1-2)	13 (72.2)
bad (3-5)	5 (27.8)

**Neuropsychological testing** n (%)	4 (22.2)
memory deficit	4 (100)
concentration deficit	3 (75)
executive function deficit	2 (50)

AC: arachnoid cyst; mRS: modified Rankin Scale.

Supratentorial ACs were present in 12 patients (66.7%), with a mean preoperative volume of 81.2 ± 44.3 cm^3^, most commonly located in the convexity (n = 8, 66.7%) or middle cranial fossa (n = 3, 25%). The middle fossa ACs were classified as Galassi Grade 2 in one case and Grade 3 in two cases. Infratentorial ACs were found in 6 patients (33.3%), predominantly in the retrocerebellar region (n = 4, 66.7%), with a mean volume of 22.1 ± 11.9 cm^3^ (Table 1). No intracystic hemorrhage, hygroma, or restricted diffusion was observed on preoperative imaging.

Fourteen patients (77.8%) were treated by endoscopic fenestration, 2 of which (14.3%) received a ventricular catheter connected to a rickham reservoir. 3 patients (16.7%) underwent microsurgical fenestration, and one patient (5.6%) received a CP shunt using a Codman–Hakim shunt system with a programmable pressure valve, initially set at 120 mmHg, placed without neuronavigation. Two patients had a history of prior AC surgery at external institutions and underwent revision procedures at our center due to symptom recurrence. Both patients reported recurring and progressively worsening headaches that differed from those experienced during the initial postoperative period, leading to a significant reduction of the quality of life. In one case, the severity of symptoms resulted in an inability to work.

### 3.2 Clinical and neurocognitive outcomes

**Fig. 3:**
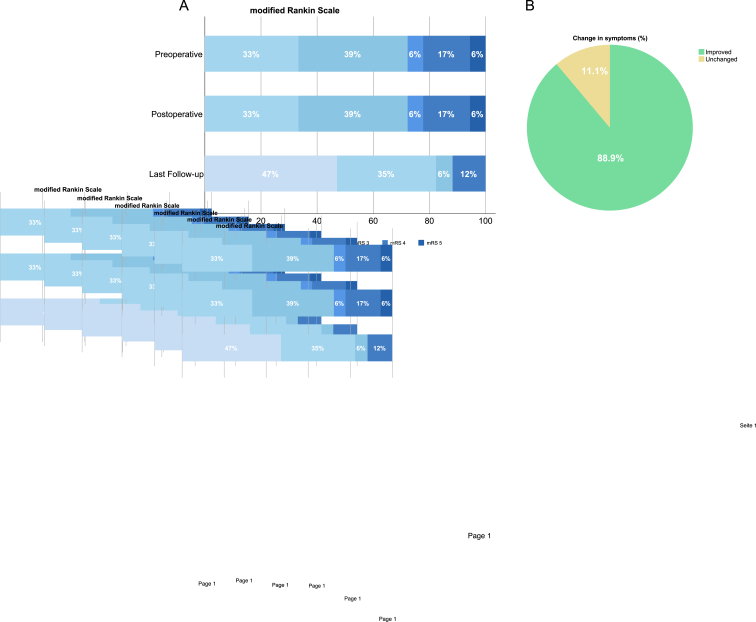
mrS and symptom change after surgical treatment A) Distribution of modified Rankin Scale (mRS) scores at baseline, postoperatively, and at last follow-up. Over time, a shift toward lower mRS scores was observed, with an increasing proportion of patients achieving mRS 0–1. B) Change in symptoms at last follow-up: 88.9% of patients reported improvement, while 11.1% remained unchanged.

At the last follow-up (mean 2.9 ± 2.4 years), 17 of 18 patients (94.4%) reported an improvement in their presenting symptoms, while one patient (5.6%) remained clinically unchanged (Table 2, Fig. 3). This patient had an infratentorial AC located in the cerebellopontine angle and underwent microsurgical fenestration as a revision procedure after prior surgery at an external institution. The patient developed postoperative ataxia due to a focal ischemia adjacent to the craniotomy site, as confirmed by early postoperative CT imaging.

**Table 2:** Clinical outcomes after surgical treatment.

	Patients (n = 18)
**Clinical presentation at last FU**	
Change in symptoms n (%)	
improved	17 (94.4)
unchanged	1 (5.6)
worse	0

**Symptom** n (%)	
none	12 (66.7)
headache	3 (16.7)
ataxia	1 (5.6)
seizures	1 (5.6)
	
**Post-operative mRS** (median (range))	1 (0-1)
mRS grouped n (%)	
good (1-2)	16 (88.9)
bad (3-5)	2 (11.1)

**Neuropsychological deficits at last FU** n (%)	n = 4
improved	4 (100)
unchanged	0
worse	0

**Complications** n (%)	7 (38.9)
**Transient complications** (resolved by last FU)	6 (33.3)
CSF leakage	3 (16.7)
wound infection	1 (5.6)
new neurological deficit	2 (11.1)
**Persistent complications** (persisting at last FU)	1 (5.6)
persisting new neurological deficit	1 (5.6)

**Reoperation** n (%)	3 (16.7)
Reoperation due to complication n (%)	
wound infection	1 (5.6)
malresorptive hydrocephalus	1 (5.6)
Reoperation due to insufficient fenestration n (%)	1 (5.6)

mRS: modified Rankin Scale; CSF: cerebrospinal fluid, FU: follow up.

Most patients (n = 12, 66.7%) were asymptomatic at follow-up, whereas 5 patients (27.8%) reported residual but improved symptoms, including headache (n = 3, 17.6%), gait disturbance (n = 1, 5.6%) and seizures (n = 1, 5.6%). Functional outcomes were favorable, with a median mRS score of 1 (0-1) at last follow-up (Table 2, Fig. 3).

All 4 patients, who underwent formal neuropsychological testing, demonstrated measurable postoperative improvement. In 2 patients, cognitive function normalized, with both achieving a maximum MMSE score of 30/30 points (from preoperative scores of 28/30 and 22/30, respectively). The other 2 patients showed improvement in the testing of the previously identified deficits in specialized neuropsychological examinations; the deficits persisted in mild form without functional impact (Table 2).

Clinical improvement was observed across all treatment modalities and cyst locations, except for the one case described above.

### 3.3 Radiological outcomes

In our study, volumetric assessment was performed using CT imaging in three patients preoperatively and in five patients postoperatively, whereas MRI-based volumetry was utilized in the remaining patients. Cyst volume decreased in all patients, with a mean relative reduction of 29.4 ± 25.3% on postoperative imaging. Supratentorial ACs, which had a higher preoperative volume, showed a higher early volume reduction (34.9 ± 22.5%) compared to infratentorial ACs (18.4 ± 27%).

Among treatment modalities, microsurgical fenestration was associated with the highest early reduction in cyst volume (62.2 ± 31.3%), compared to endoscopic fenestration (22.6 ± 18.4%) and CP shunt placement (25.4%).

**Fig. 4:**
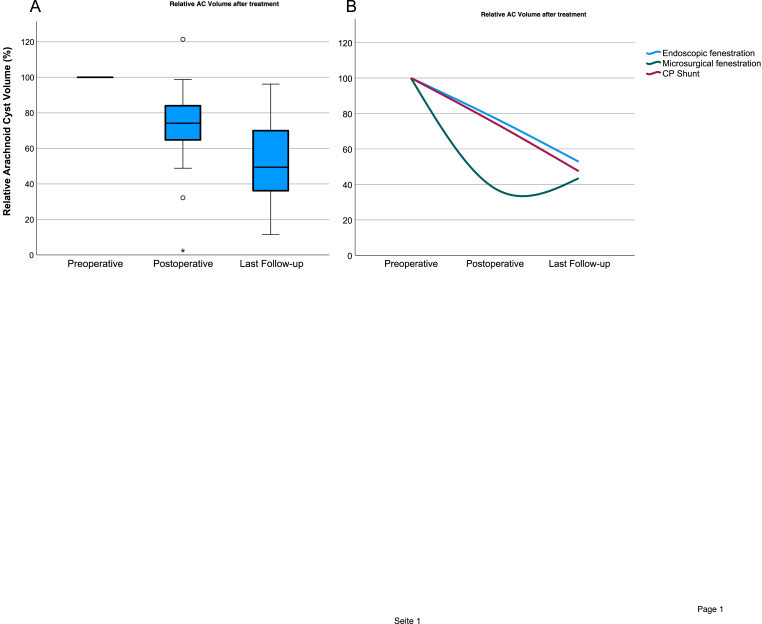
Relative volume change of AC after surgical treatment **A)** Boxplots illustrating the relative AC volume change (percent of preoperative volume) at three time points: preoperatively, postoperatively, and at last follow-up. Median, interquartile ranges, and outliers are displayed. **B)** Line graph showing the mean relative AC volume reduction over the study timeline, stratified by surgical technique and location.

At the last follow-up, the overall mean relative volume reduction was 44.5 ± 30.4% and only non-significant differences in volume reduction were observed between treatment modalities (Fig. 4). Cyst volume reduction was not correlated with the observed symptom improvement (OR 1.02, [95% CI 0.95 - 1.08]; p = 0.637).

### 3.4 Complications

No intraoperative complications occurred. Postoperative complications were observed in 7 patients (38.9%), of which 6 (33.3%) were transient. The most frequent complication was CSF leakage from the surgical wound (n = 3, 16.7%), which occurred in 2 patients treated with endoscopic fenestration and one patient treated microsurgically. All cases were managed conservatively without long-term sequelae. Transient neurological deficits were observed in two patients (11.1%): one patient in the endoscopic group (temporo-occipital AC) developed a transient speech disturbance, and one patient in the microsurgical group (convexity AC) experienced transient weakness of the toes. Additionally, one patient (5.6%) in the endoscopic group developed a wound infection with ventriculitis requiring surgical revision and intravenous antibiotic treatment for 20 days.

The only permanent complication was a new neurological deficit with persistent ataxia in the patient with a cerebellopontine angle AC who underwent revision microsurgical fenestration, as described above.

When stratified by treatment modality, complications occurred in 4 of 14 patients (28.6%) treated endoscopically and all 3 patients (100%) treated microsurgically. No complications were observed in the patient treated with primary CP shunting. Regarding cyst location, complications occurred in 4 of 12 patients (33.3%) with supratentorial ACs and in 3 of 6 patients (50.0%) with infratentorial ACs.

Surgical treatment of complications was required in 2 (11.1%) patients, the patient with wound revision after infection mentioned above, and one patient with a post-operative malresorptive hydrocephalus being treated with ventriculoperitoneal shunt placement. Preoperatively, this patient presented with mildly enlarged ventricles due to compression of the fourth ventricle by the retrocerebellar AC. Initial postoperative imaging demonstrated improvement in both the compression and the width of the fourth ventricle. However, one month after surgery, the patient presented with a subgaleal CSF collection and CSF leakage from the wound. Following initial management with direct suture closure of the CSF leakage site and the placement of a lumbar drain for 3 days, the indication for shunt placement due to malresorptive hydrocephalus was set. After ventriculoperitoneal shunt placement and the patient exhibited no further radiological or clinical signs of impaired cerebrospinal fluid circulation. No mortality was observed.

### 3.5 Patient-reported outcomes

Of the 18 surgically treated patients, 9 (50%) participated in the PROMs questionnaire. Overall, subjective health perception and quality of life showed improvement in most patients. Subjective health perception improved in 7 (77.8%) and quality of life in 8 (88.9%) patients; all patients attributed these improvements to the surgical intervention rather than unrelated life events (Table 4) (see Table 5).

**Table 3:** Radiological outcomes after surgical treatment.

	Patients (n = 18)
**Cyst volume change** (% (±SD))	
pre-OP to 1d post-surgery	−29.4 (25.3)
post OP to last FU	−44.5 (30.4)
**Radiological evaluation of drainage** n (%)	
sufficient	17 (94.4)
insufficient	1 (5.6)

FU: follow up.

**Table 4:** Patient-reported outcome measures (PROMs) after surgical treatment.

	Participants (n = 9)
**Employment status**	
Pre-operative n (%)	
employed	7 (77.8)
retired	1 (11.1)
on sick leave	1 (11.1)
Postoperative n (%)	
employed	8 (88.9)
retired	1 (11.1)
on sick leave	0

**Time to return to work after surgery** in weeks (median (range))	4 (2-16)
**Satisfaction with surgery n (%)**	
Surgery met patient expectation	8 (88.9)
Surgery did not meet patient expectation	1 (11.1)
Patient would recommend surgery	8 (88.9)
Patient would not recommend surgery	1 (11.1)
**Health perception**	
direction of change n (%)	
improved	7 (77.8)
unchanged	1 (11.1)
worse	1 (11.1)

**Quality of life**	
direction of change n (%)	
improved	8 (88.9)
unchanged	0
worse	1 (11.1)

**Headache**	
direction of change n (%)	
improved	5 (55.6)
unchanged	0
worse	0
no headache pre-operatively	4 (44.4)

**Table 5:** PROM: Subjective patient assessment of cognitive functions after surgical treatment.

	Participants (n = 9)
**Memory**	
direction of change n (%)	
improved	6 (66.7)
unchanged	1 (11.1)
worse	2 (22.2)

**Mathematic ability**	
direction of change n (%)	
improved	5 (55.6)
unchanged	3 (33.3)
worse	1 (11.1)

**Executive function**	
direction of change n (%)	
improved	4 (44.4)
unchanged	4 (44.4)
worse	1 (11.1)

**Concentration**	
direction of change n (%)	
improved	7 (77.8)
unchanged	0
worse	2 (22.2)

**Ability to perform daily cognitive tasks**	
direction of change n (%)	
improved	5 (55.6)
unchanged	2 (22.2)
worse	2 (22.2)

**Ability to perform daily physical tasks**	
direction of change n (%)	
improved	1 (11.1)
unchanged	6 (66.7)
worse	2 (22.2)

**Ability to fulfil social roles**	
direction of change n (%)	
improved	4 (44.4)
unchanged	5 (55.6)
worse	0

**Fig. 5:**
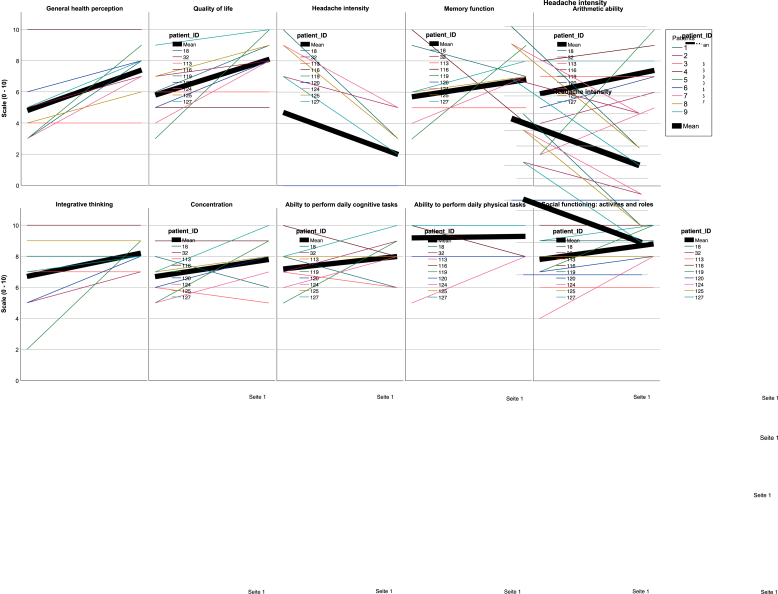
Patient-reported outcome measures (PROMs) after surgical treatment Individual patient trajectories (colored lines) and mean values (bold black line) are shown for different PROM domains assessed on a 0–10 scale in the telephone survey. Outcomes included general health perception, quality of life, headache intensity, memory function, arithmetic ability, integrative thinking, concentration, ability to perform daily cognitive tasks, ability to perform daily physical tasks, and social functioning. Postoperatively, most patients demonstrated improvements in general health perception, quality of life, cognitive and functional domains, with a reduction in headache intensity.

Among patients who were employed before surgery, all returned to work after a median of 4 weeks (Table 3). Additionally, one previously unemployed patient was able to work again after symptom resolution due to surgery. Self-reported cognitive functioning improved in most domains postoperatively (Table 4, Fig. 5). In the three patients who underwent both objective neuropsychological testing and the PROMs survey, subjective cognitive assessment of the affected domains aligned with the objectively documented deficits as well as with their postoperative improvement.

Satisfaction and perceived benefits were observed across all treatment modalities and cyst locations. Among the nine patients who completed the PROMs questionnaire, 6 (66.7%) had undergone endoscopic fenestration, 2 (22.2%) microsurgical fenestration, and 1 (11.1%) CP shunting. Apart from health perception, which was rated as higher by endoscopically treated patients, the reported subjective improvement in subjective cognitive outcome and quality of life showed no notable differences between surgical techniques. No apparent differences were observed between patients with supratentorial (n = 6) and infratentorial (n = 3) ACs.

Eight patients (88.9%) stated that the surgery met their expectations and reported that they would recommend the procedure to others (Table 3).

## 4 Discussion

This retrospective case series provides a multidimensional evaluation of clinical, radiological, neurocognitive, and patient-reported outcome data from 18 adults undergoing surgical treatment for symptomatic intracranial AC. Symptom improvement was achieved in 94.4% of patients, with a mean cyst volume reduction of 44.5% and no surgical mortality. Objective neurocognitive deficits improved in all formally assessed cases, and 88.9% of respondents expressed satisfaction with the surgical outcome, including full occupational reintegration. These findings support the potential benefit of surgery in selected patients and underscore the value of multidimensional outcome evaluation in this heterogeneous population.

### 4.1 Surgical indication and techniques

Of the 2537 adult patients with radiologically diagnosed intracranial AC at our institution during the study period, only 0.71% underwent surgery. While some may have been referred elsewhere, this low rate reflects the restrictive criteria to determine surgical indication at our center, where surgery is reserved for carefully selected patients with persistent or progressive symptoms. The mean interval of 16.6 ± 28.5 months between diagnosis and surgery further underscores the individualized decision-making process, often requiring detailed longitudinal assessment to confirm a causal relationship between symptom and cyst and exclude alternative causes.

However, this low surgical rate may also suggest that subtle, yet clinically relevant, neurocognitive symptoms remain underrecognized. Until recent years, formal neuropsychological testing was not routinely incorporated into the diagnostic workup at our institution. As a result, some patients with mild cognitive impairments may not have been identified as surgical candidates. Notably, the mean age in our case series was 51 years, indicating that most patients were in the midst of their working lives, where even subtle cognitive impairments may significantly affect occupational performance and quality of life. Based on our findings and in line with prior reports, we advocate for systematic neurocognitive and psychological assessment in patients with AC to avoid undertreatment and ensure appropriate patient selection (Spansdahl and Solheim, 2007; Chen et al., 2016; Torgersen et al., 2010; Di Rocco, 2010; Moss et al., 2019).

Endoscopic fenestration has become the preferred surgical technique at our institution, favored for its minimally invasive nature, shorter recovery time, and avoidance of foreign material. The goal is to establish communication between the cyst and subarachnoid space, thereby equalizing intracystic and subarachnoid pressures, and reducing cyst volume and mass effect. This physiological rationale is supported by studies that directly measured intracystic pressure, demonstrating the existence of these pressure gradients, with clinical improvement often occurring irrespective of absolute pressure values (Kershenovich et al., 2018; Helland and Wester, 2007). In our series, the majority of patients (77.8%) were treated endoscopically, while microsurgical fenestration was reserved mainly for superficial or posterior fossa cysts (particularly cerebellopontine-angle cysts), and CP shunting was used as a secondary intervention after recurrence. Although stereotactically implanted cysto-ventricular catheters represent an alternative to CP shunting mentioned in the literature (Lackermair et al., 2024; Magnéli et al., 2021), they were not utilized in our cohort. Overall, this treatment strategy aligns with current literature (Chen et al., 2016; Wang et al., 2013).

### 4.2 Clinical and radiological outcomes

In this study, 94.4% of patients reported clinical improvement at the last follow-up. The most frequent preoperative symptoms were headache (66.7%), cognitive complaints (33.3%), and dizziness or gait disturbances (27.8%), consistent with prior reports on symptomatic ACs (Kandenwein et al., 2004; Chen et al., 2016; Kershenovich et al., 2018; Torgersen et al., 2010; Jaradat et al., 2024; Wang et al., 2013; Khan et al., 2013; Masoudi et al., 2021). Clinical improvement rates in previous studies range from 66 to 93% underscoring the potential benefit of surgery in appropriately selected patients (Rabiei et al., 2017; Kandenwein et al., 2004; Spansdahl and Solheim, 2007; Chen et al., 2016; Pradilla and Jallo, 2007; Torgersen et al., 2010; Hayes et al., 2019; Moss et al., 2019; Helland and Wester, 2007; Wang et al., 2013; Khan et al., 2013; Mørkve et al., 2016).

Subtle cognitive impairments have gained increasing attention in AC patients. Prior studies have described deficits particularly in memory, attention, and executive function, which are often underrecognized in routine clinical practice (Torgersen et al., 2010; Gjerde et al., 2019). In our case series, formal neuropsychological assessments confirmed mild but measurable preoperative deficits, all of which improved postoperatively, some normalizing entirely. These findings support existing literature suggesting AC-associated cognitive deficits do exist and are often reversible through surgical treatment, even after prolonged symptom duration (Raeder et al., 2005; Wester, 2008).

The overall postoperative complication rate was 38.9%, however the majority of complications were transient (33.3%) and had fully resolved by the last follow-up. In the endoscopic group, 4 patients (28.6%) experienced transient complications—most commonly self-limiting CSF leakage (n = 2), managed conservatively without sequelae. In the microsurgical group, complications occurred in all three patients, including one case of ischemic ataxia, one transient motor deficit, and one case of postoperative hydrocephalus requiring shunt placement. The complication rates of endoscopic surgery and shunt placement are consistent with previously reported complication rates of 15-49%, depending on the surgical technique, cyst location, and patient selection (Spansdahl and Solheim, 2007; Chen et al., 2016). In contrast, the complication rate of microsurgical complication is higher than previously reported. Although the small sample size across the different surgical approaches limits definitive comparisons, this observation may tentatively suggest a higher morbidity profile associated with microsurgical techniques, possibly reflecting the more invasive nature of the approach or the increased complexity of selected cases, as microsurgical fenestration was primarily chosen for cysts that were not accessible endoscopically. This is consistent with the existing literature, which indicates that endoscopic fenestration is generally associated with lower complication rates and reduced procedure-related morbidity compared with microsurgical approaches or primary shunt placement (Chen et al., 2016; Wang et al., 2013; Khan et al., 2013; Fernández Molina, 2013).

Radiological follow-up showed a mean cyst volume reduction of 44.5%, slightly lower than previously reported ranges of 58-74% (Kandenwein et al., 2004). Whether this reduction correlates with clinical outcome remains uncertain. While some studies have suggested a positive association between cyst volume reduction and symptom improvement, others report no clear association (Rabiei et al., 2017; Kandenwein et al., 2004; Di Rocco, 2010; Helland and Wester, 2007; Khan et al., 2013; Fernández Molina, 2013; Koch et al., 1998; Erman et al., 2004; Gui et al., 2011). In our series, no correlation between cyst volume reduction and symptom improvement was observed (OR 1.02, [95% CI 0.95 - 1.08]; p = 0.637), however due to our small cohort size, this correlation should be interpreted as exploratory. Therefore, with the prognostic relevance of cyst volume reduction remaining uncertain, our results suggest that pressure equalization between the intracystic and intracranial compartments may be sufficient even without achieving a substantial reduction in cyst volume. Further, this highlights that outcome evaluation should include clinical status, neurocognitive function, and patient-reported metrics for a more comprehensive assessment of treatment effectiveness.

### 4.3 Patient-reported outcomes

To our knowledge, this is the first case series to use a comprehensive, multidimensional questionnaire for AC patients. In the absence of a validated, AC-specific PROM instrument, we developed a structured telephone-based assessment covering key domains such as perceived cognitive function, pain, sleep quality, functional independence, occupational reintegration, and overall satisfaction with the surgical treatment. Among these domains, improvements were most frequently reported in perceived quality of life, cognitive functioning, headache intensity, and return to work. These subjective benefits complement the clinical and neurocognitive improvements observed in our series and suggest that surgical treatment of symptomatic ACs may result in meaningful improvements in daily functioning and psychosocial well-being. Importantly, nearly 90% of patients indicated that the procedure met or exceeded their expectations and that they would recommend surgery to others. This is consistent with a prior study reporting a 91.5% satisfaction rate in long-term postoperative follow-up (Moss et al., 2019). Such high levels of satisfaction underscore that patients' perceptions of surgical benefit extend beyond symptom relief, aligning closely with broader quality-of-life priorities. While generic PROMs such as the SF-36 and the Glasgow Benefit Inventory have previously demonstrated postoperative benefits in this population, they may not fully capture the specific and multifaceted impact of AC surgery on patients’ lives (Moss et al., 2019; Mørkve et al., 2016). Our findings highlight the potential value of individualized, procedure-specific PROM instruments to more accurately assess outcomes in this population. Future studies should aim to develop and validate AC-specific PROM tools to improve outcome assessment and support patient-centered care.

### 4.4 Limitations

This study has several limitations. Its retrospective design precluded the collection of preoperative baseline PROMs, and the delayed follow-up may have introduced recall bias and confounding factors such as aging or other unrelated life events. Two patients explicitly reported difficulty in retrospectively assessing cognitive changes. Due to the absence of a validated AC-specific patient satisfaction instrument, satisfaction was assessed based on individual expectations, which are subject to interpersonal differences. Additionally, only 50% of patients completed the PROMs assessment, introducing response bias. Neuropsychological testing was not performed systematically across all patients, limiting the comparability of cognitive outcomes. A standardized assessment protocol has since been implemented at our institution to address this. Furthermore, volumetric assessment of arachnoid cysts was based on routinely acquired clinical imaging, resulting in heterogeneity regarding imaging modality and sequence availability. High-resolution MRI sequences (e.g., CISS) were not consistently available across the cohort. To preserve longitudinal consistency within individual patients, both CT and MRI studies were included when necessary. However, as CT is inherently less accurate than MRI for delineating cyst membranes, this may have reduced the precision of volumetric measurements. Lastly, the small sample size limits generalizability and precludes multivariate analysis. This reflects the rarity of surgically treated ACs and underscores the need for prospective, multicenter studies with standardized outcome measures to strengthen the evidence base.

## 5 Conclusion

In well-selected patients, surgical treatment of symptomatic AC is associated with clinical and neurocognitive improvement, high patient satisfaction, and a low rate of permanent morbidity. Patient-reported outcomes further support the positive impact of surgical treatment on quality of life and functional recovery. These findings highlight the potential value of surgery in this population—particularly when treatment decisions are guided by comprehensive clinical and neuropsychological assessment.

## Declaration of competing interest

The authors declare that they have no known competing financial interests or personal relationships that could have appeared to influence the work reported in this paper.
