# Supplementary material for: Surgical treatment for arachnoid cysts in adults: Clinical, radiological, neurocognitive and patient-reported outcomes
Source: Brain Spine. 2026 May 1;6:106077. doi: 10.1016/j.bas.2026.106077 (PMC13157037; doi:10.1016/j.bas.2026.106077)
Supplement: Multimedia component 1 [file mmc1.docx]

# **Supplementary Materials**

**1.1 PROMs Questionnaire version 1.2 (translated to English)**

**Surgically treated AC PROMs - Telephone Questionnaire**

**Patient ID:**

**Date: _________________________**

**Employment Section:**

1. **What is the highest level of education you have completed?**

**Elementary school  Elementary school**

**not completed  Vocational training**

**High school diploma/vocational diploma**

**University /University of Applied Sciences**

**Other:___________________**

1. **What was your employment status before surgery?**

**Employed  Unemployed/Job seeker  Retired**

**Disability pensioner  Homemaker  On sick leave (unable to  Other:___________________ work)**

- 1. **If unable to work, were you able to return to work after surgery?**

**yes  no  unknown/not available**

- - 1. **If no: What prevents you from returning to your previous occupation:________________________________________________**
    2. **Would you like to return to your previous occupation?**

**yes  no  unknown/not available**

- - 1. **If you are currently unable to work in your previous occupation, can you work in another occupation instead?**

**yes  no  unknown/not available**

- 1. **If employed, were you able to return to your previous occupation after your surgery?**

**yes  no  unknown/not available**

- - 1. **How long after the surgery did it take before you were able to return to your previous occupation (weeks): __________________**
    2. **Was there a change in your work hours after the surgery?**

**yes  no  unknown/not available**

- - 1. **Were there any changes to your job duties after the surgery?**

**yes  no  unknown/not available**

1. **What is your current employment status?**

**Employed  Unemployed/Job seeker  Retired**

**Disability pensioner  Homemaker  On sick leave (unable to  Other:___________________ work)**

**General Perception of Health**

1. **On a scale of 1 to 10 (1 being the worst, 10 being the best), how would you describe your current general state of health?**

| **1** | **2** | **3** | **4** | **5** | **6** | **7** | **8** | **9** | **10** |
| --- | --- | --- | --- | --- | --- | --- | --- | --- | --- |

1. **How would you describe your health before the surgery, on a scale of 1 to 10 (1 being the worst, 10 being the best)?**

| **1** | **2** | **3** | **4** | **5** | **6** | **7** | **8** | **9** | **10** |
| --- | --- | --- | --- | --- | --- | --- | --- | --- | --- |

1. **To what extent do each of the following statements apply to you?**

**☐ Much better ☐ Somewhat better ☐ Unchanged**

**☐ Somewhat worse ☐ Much worse**

1. **To what extent does each of the following statements apply to you?**
   1. **I seem to get sick more easily than others.**

**Definitely true  Mostly true  Don’t know
 Mostly false  Definitely false**

- 1. **I am just as healthy as everyone else.**

**Definitely true  Mostly true  Don’t know
 Mostly false  Definitely false**

- 1. **I expect my health to decline.**

**Definitely true  Mostly true  Don’t know
 Mostly false  Definitely false**

- 1. **I enjoy excellent health.**

**Definitely true  Mostly true  Don’t know
 Mostly false  Definitely false**

**Cognitive Functions Section**

1. **How would you rate your memory in general:**

| **Before surgery** | **Good** | **Poor** |  | **Don’t know** |
| --- | --- | --- | --- | --- |
| **After surgery** | **Better than before surgery** | **Worse than before surgery** | **Unchanged from before surgery** | **Don’t know** |

**At this time, on a scale of 1 to 10 (1 being worst, 10 being best)?**

| **1** | **2** | **3** | **4** | **5** | **6** | **7** | **8** | **9** | **10** |
| --- | --- | --- | --- | --- | --- | --- | --- | --- | --- |

**Before surgery, on a scale of 1 to 10 (1 being worst, 10 being best)?**

| **1** | **2** | **3** | **4** | **5** | **6** | **7** | **8** | **9** | **10** |
| --- | --- | --- | --- | --- | --- | --- | --- | --- | --- |

1. **How would you rate your short-term memory:**

| **Before surgery** | **Good** | **Poor** |  | **Don’t know** |
| --- | --- | --- | --- | --- |
| **After surgery** | **Better than before surgery** | **Worse than before surgery** | **Unchanged from before surgery** | **Don’t know** |

**At this time, on a scale of 1 to 10 (1 being worst, 10 being best)?**

| **1** | **2** | **3** | **4** | **5** | **6** | **7** | **8** | **9** | **10** |
| --- | --- | --- | --- | --- | --- | --- | --- | --- | --- |

**Before surgery, on a scale of 1 to 10 (1 being worst, 10 being best)?**

| **1** | **2** | **3** | **4** | **5** | **6** | **7** | **8** | **9** | **10** |
| --- | --- | --- | --- | --- | --- | --- | --- | --- | --- |

1. **How would you rate your long-term memory:**

| **Before surgery** | **Good** | **Poor** |  | **Don’t know** |
| --- | --- | --- | --- | --- |
| **After surgery** | **Better than before surgery** | **Worse than before surgery** | **Unchanged from before surgery** | **Don’t know** |

**At this time, on a scale of 1 to 10 (1 being worst, 10 being best)?**

| **1** | **2** | **3** | **4** | **5** | **6** | **7** | **8** | **9** | **10** |
| --- | --- | --- | --- | --- | --- | --- | --- | --- | --- |

**Before surgery, on a scale of 1 to 10 (1 being worst, 10 being best)?**

| **1** | **2** | **3** | **4** | **5** | **6** | **7** | **8** | **9** | **10** |
| --- | --- | --- | --- | --- | --- | --- | --- | --- | --- |

1. **Are there/have there been situations where your memory works better or worse?**

| **Before the surgery** | **Yes** | **No** | **Don’t know** |
| --- | --- | --- | --- |
| If yes, in which  situations better? |  | | |
| If yes, in which  situations worse? |  | | |
| **At this point in time** | **Yes** | **No** | **Don’t know** |
| If yes, in which  situations better? |  | | |
| If yes, in which  situations worse? |  | | |

1. **How would you rate your math skills:**

| **Before surgery** | **Good** | **Poor** |  | **Don’t know** |
| --- | --- | --- | --- | --- |
| **After surgery** | **Better than before surgery** | **Worse than before surgery** | **Unchanged from before surgery** | **Don’t know** |

**At this time, on a scale of 1 to 10 (1 being worst, 10 being best)?**

| **1** | **2** | **3** | **4** | **5** | **6** | **7** | **8** | **9** | **10** |
| --- | --- | --- | --- | --- | --- | --- | --- | --- | --- |

**Before surgery, on a scale of 1 to 10 (1 being worst, 10 being best)?**

| **1** | **2** | **3** | **4** | **5** | **6** | **7** | **8** | **9** | **10** |
| --- | --- | --- | --- | --- | --- | --- | --- | --- | --- |

1. **How would you rate your ability to identify connections and make links?**

| **Before surgery** | **Good** | **Poor** |  | **Don’t know** |
| --- | --- | --- | --- | --- |
| **After surgery** | **Better than before surgery** | **Worse than before surgery** | **Unchanged from before surgery** | **Don’t know** |

**At this time, on a scale of 1 to 10 (1 being worst, 10 being best)?**

| **1** | **2** | **3** | **4** | **5** | **6** | **7** | **8** | **9** | **10** |
| --- | --- | --- | --- | --- | --- | --- | --- | --- | --- |

**Before surgery, on a scale of 1 to 10 (1 being worst, 10 being best)?**

| **1** | **2** | **3** | **4** | **5** | **6** | **7** | **8** | **9** | **10** |
| --- | --- | --- | --- | --- | --- | --- | --- | --- | --- |

1. **How would you rate your ability to concentrate?**

| **Before surgery** | **Good** | **Poor** |  | **Don’t know** |
| --- | --- | --- | --- | --- |
| **After surgery** | **Better than before surgery** | **Worse than before surgery** | **Unchanged from before surgery** | **Don’t know** |

**At this time, on a scale of 1 to 10 (1 being worst, 10 being best)?**

| **1** | **2** | **3** | **4** | **5** | **6** | **7** | **8** | **9** | **10** |
| --- | --- | --- | --- | --- | --- | --- | --- | --- | --- |

**Before surgery, on a scale of 1 to 10 (1 being worst, 10 being best)?**

| **1** | **2** | **3** | **4** | **5** | **6** | **7** | **8** | **9** | **10** |
| --- | --- | --- | --- | --- | --- | --- | --- | --- | --- |

1. **How would you rate your ability to handle everyday tasks?**

| **Before surgery** | **Good** | **Poor** |  | **Don’t know** |
| --- | --- | --- | --- | --- |
| **After surgery** | **Better than before surgery** | **Worse than before surgery** | **Unchanged from before surgery** | **Don’t know** |

**At this time, on a scale of 1 to 10 (1 being worst, 10 being best)?**

| **1** | **2** | **3** | **4** | **5** | **6** | **7** | **8** | **9** | **10** |
| --- | --- | --- | --- | --- | --- | --- | --- | --- | --- |

**Before surgery, on a scale of 1 to 10 (1 being worst, 10 being best)?**

| **1** | **2** | **3** | **4** | **5** | **6** | **7** | **8** | **9** | **10** |
| --- | --- | --- | --- | --- | --- | --- | --- | --- | --- |

**Quality of Life Section**

1. **Are you independent in your daily life?**

**Yes  No**

- 1. **If no, do you think this is related to surgery?**

**yes  no  unknown/not applicable**

1. **Were you independent in your daily life before surgery?**

**Yes  No**

1. **If applicable, which daily activities can you no longer perform completely on your own?**

| **Shopping** | **Cooking** | **Eating & drinking** | **Laundry** |
| --- | --- | --- | --- |
| **Moving around independently at home (mobility)** | **Taking medication** | **Financial transactions** | **Personal hygiene (bathing or getting dressed)** |
| **Strenuous activities (exercising)** | **Household chores (e.g., repairs, cleaning the apartment/house)** | **Scheduling and keeping appointments** | **Walking more than one block** |

**Were some of these abilities not possible before the surgery?**

**Yes  No**

**If yes: which: ___________________________**

1. **On a scale of 1 to 10 (1 = not at all independent, 10 = completely independent), to what extent can you currently perform your daily physical activities (such as walking, climbing stairs, carrying groceries, or moving a chair) on your own?**

| **1** | **2** | **3** | **4** | **5** | **6** | **7** | **8** | **9** | **10** |
| --- | --- | --- | --- | --- | --- | --- | --- | --- | --- |

**Before the surgery?**

| **1** | **2** | **3** | **4** | **5** | **6** | **7** | **8** | **9** | **10** |
| --- | --- | --- | --- | --- | --- | --- | --- | --- | --- |

1. **On a scale of 1 to 10 (1 being the worst and 10 being the best), how well are you currently able to carry out your usual social activities and roles? (This includes activities at home, at work, and in your wider social circle. It also refers to responsibilities as a parent, child, spouse, employee, friend, etc.)**

| **1** | **2** | **3** | **4** | **5** | **6** | **7** | **8** | **9** | **10** |
| --- | --- | --- | --- | --- | --- | --- | --- | --- | --- |

**Before the surgery?**

| **1** | **2** | **3** | **4** | **5** | **6** | **7** | **8** | **9** | **10** |
| --- | --- | --- | --- | --- | --- | --- | --- | --- | --- |

1. **On a scale of 1 to 10 (1 = not at all, 10 = very much), to what extent have your physical health or mental health issues affected your normal interactions with family members, friends, neighbors, or acquaintances over the past 4 weeks?**

| **1** | **2** | **3** | **4** | **5** | **6** | **7** | **8** | **9** | **10** |
| --- | --- | --- | --- | --- | --- | --- | --- | --- | --- |

**Before the surgery?**

| **1** | **2** | **3** | **4** | **5** | **6** | **7** | **8** | **9** | **10** |
| --- | --- | --- | --- | --- | --- | --- | --- | --- | --- |

1. **On a scale of 1 to 10 (with 1 being the worst and 10 being the best), how would you rate your current satisfaction with your social activities and relationships?**

| **1** | **2** | **3** | **4** | **5** | **6** | **7** | **8** | **9** | **10** |
| --- | --- | --- | --- | --- | --- | --- | --- | --- | --- |

**Before the surgery?**

| **1** | **2** | **3** | **4** | **5** | **6** | **7** | **8** | **9** | **10** |
| --- | --- | --- | --- | --- | --- | --- | --- | --- | --- |

1. **Is your quality of life after the surgery:**

**Better  Same  Worse  Don’t know**

**If “better” or “worse,” what has changed?**

**_____________________________________________________________________**

1. **If worse: Do you think the decline in your quality of life is related to the surgery?**

**Yes  No  Don’t know**

1. **If better: Do you think the improvement in your quality of life is related to the surgery?**

**Yes No  Don’t know**

1. **On a scale of 1 to 10 (with 1 being the worst and 10 being the best), how would you rate your quality of life today?**

| **1** | **2** | **3** | **4** | **5** | **6** | **7** | **8** | **9** | **10** |
| --- | --- | --- | --- | --- | --- | --- | --- | --- | --- |

**Before the surgery?**

| **1** | **2** | **3** | **4** | **5** | **6** | **7** | **8** | **9** | **10** |
| --- | --- | --- | --- | --- | --- | --- | --- | --- | --- |

**Emotions Section**

How often have you experienced the following emotional states in the past 4 weeks:

| **Full of pep** | **Always** | **Most of the time** | **Quite often** | **Sometimes** | **Rarely** | **Never** |
| --- | --- | --- | --- | --- | --- | --- |
| **Very nervous** | **Always** | **Most of the time** | **Quite often** | **Sometimes** | **Rarely** | **Never** |
| **Depressed** | **Always** | **Most of the time** | **Quite often** | **Sometimes** | **Rarely** | **Never** |
| **Calm and peaceful** | **Always** | **Most of the time** | **Quite often** | **Sometimes** | **Rarely** | **Never** |
| **Full of energy** | **Always** | **Most of the time** | **Quite often** | **Sometimes** | **Rarely** | **Never** |
| **Downhearted and blue** | **Always** | **Most of the time** | **Quite often** | **Sometimes** | **Rarely** | **Never** |
| **Won out** | **Always** | **Most of the time** | **Quite often** | **Sometimes** | **Rarely** | **Never** |
| **Aggressive** | **Always** | **Most of the time** | **Quite often** | **Sometimes** | **Rarely** | **Never** |
| **Happy** | **Always** | **Most of the time** | **Quite often** | **Sometimes** | **Rarely** | **Never** |
| **Tired** | **Always** | **Most of the time** | **Quite often** | **Sometimes** | **Rarely** | **Never** |

**Pain Section**

1. **How severe was your pain:**

| **Before surgery** | **None** | **Very mild** | **Mild** | **Moderate** | **Severe** | **Very severe** |
| --- | --- | --- | --- | --- | --- | --- |
| **After surgery** | **None** | **Very mild** | **Mild** | **Moderate** | **Severe** | **Very severe** |

**Compared to before the surgery, how is your pain now?**

**☐ Better ☐ The same ☐ Worse ☐ Don’t know/no pain before**

1. **At this time, on a scale of 1 to 10 (1 = no pain, 10 = very severe pain)?**

| **1** | **2** | **3** | **4** | **5** | **6** | **7** | **8** | **9** | **10** |
| --- | --- | --- | --- | --- | --- | --- | --- | --- | --- |

**Before surgery?**

| **1** | **2** | **3** | **4** | **5** | **6** | **7** | **8** | **9** | **10** |
| --- | --- | --- | --- | --- | --- | --- | --- | --- | --- |

1. **To what extent has the pain interfered with your ability to perform your daily activities at home and at work over the past 4 weeks?**

**Not at all  A little  Moderately  Quite a bit
 Extremely**

**Sleep Section**

How would you rate the quality of your sleep:

| **Before surgery** | **Good** | **Poor** |  | **Don’t know** |
| --- | --- | --- | --- | --- |
| **After surgery** | **Better than before surgery** | **Worse than before surgery** | **Unchanged from before surgery** | **Don’t know** |

**At this time, on a scale of 1 to 10 (1 being worst, 10 being best)?**

| **1** | **2** | **3** | **4** | **5** | **6** | **7** | **8** | **9** | **10** |
| --- | --- | --- | --- | --- | --- | --- | --- | --- | --- |

**Before surgery, on a scale of 1 to 10 (1 being worst, 10 being best)?**

| **1** | **2** | **3** | **4** | **5** | **6** | **7** | **8** | **9** | **10** |
| --- | --- | --- | --- | --- | --- | --- | --- | --- | --- |

**Conclusion**

**Would you recommend this surgery?**

**Yes  No  Don’t know**

**Patient Satisfaction Index Score**

| **Surgery met my expectations** | **1** |
| --- | --- |
| **Surgery improved my condition enough so that I would go through it again for the same outcome** | **2** |
| **Surgery helped me but I would not go through it again for the same outcome** | **3** |
| **I am the same or worse compared to after surgery** | **4** |

**Other comments:**

**___________________________________________________________________________**

**1.2 PROMs Questionnaire version 1.2 (Original German version)**

**Surgically treated AC PROMS - Telefonischer Fragebogen**

**Patienten ID:**

**Datum: _________________________**

**Sektion Beschäftigung:**

1. **Was ist Ihre höchste abgeschlossene Ausbildung?**

**Kein Grundschulabschluss  Grundschulabschluss  Lehrabschluss**

**Matur/Berufsmatur  Universität/Fachhochschule**

**Anderes:___________________**

1. **Was war Ihre Berufsstellung vor der Operation?**

**Berufstätig  Arbeitslos/Auf Arbeitssuche  Rentner  IV Rentner**

**Hausfrau/Mann  Krankgeschrieben (nicht arbeitsfähig)**

**Anderes:___________________**

- 1. **Falls nicht arbeitsfähig, können Sie nach Ihrer Operation wieder einen Beruf ausüben?**

**ja  nein  unbekannt/n.a.**

- - 1. **Was hindert Sie daran Ihren früheren Beruf wieder auszuüben:**

**__________________________________________________________**

- - 1. **Würden Sie gerne Ihren früheren Beruf wieder ausüben?**

**ja  nein  unbekannt/n.a.**

- - 1. **Können Sie, falls Sie Ihren früheren Beruf bisher nicht ausüben können, stattdessen einen anderen Beruf ausüben?**

**☐ ja ☐ nein ☐ unbekannt/n.a.**

- 1. **Falls berufstätig, konnten Sie nach Ihrer Operation Ihren früheren Beruf wieder ausüben?**

**ja  nein  unbekannt/n.a.**

- - 1. **Wie lange hat es nach der Operation gedauert, bis Sie ihren früheren Beruf wieder ausüben konnten (Wochen): __________________**
    2. **Gab es nach der Operation eine Veränderung Ihrer Arbeitszeit?**

**☐ ja ☐ nein ☐ unbekannt/n.a.**

- - 1. **Falls ja, gab es nach der Operation eine Veränderung Ihrer Arbeitsaufgaben?**

**☐ ja ☐ nein ☐ unbekannt/n.a.**

1. **Wie ist Ihre jetzige Berufsstellung?**

**Berufstätig  Arbeitslos/Auf Arbeitssuche  Rentner  IV Rentner**

**Hausfrau/Mann  Krankgeschrieben  Anderes:___________________**

**Allgemeine Gesundheitswahrnehmung**

1. **Wie würden Sie Ihren jetzigen Gesundheitszustand im Allgemeinen, auf einer Skala von 1 bis 10 (1 als Schlechtestes 10 als Bestes), beschreiben?**

| **1** | **2** | **3** | **4** | **5** | **6** | **7** | **8** | **9** | **10** |
| --- | --- | --- | --- | --- | --- | --- | --- | --- | --- |

1. **Wie würden Sie Ihren Gesundheitszustand vor der OP, auf einer Skala von 1 bis 10 (1 als Schlechtestes 10 als Bestes), beschreiben?**

| **1** | **2** | **3** | **4** | **5** | **6** | **7** | **8** | **9** | **10** |
| --- | --- | --- | --- | --- | --- | --- | --- | --- | --- |

1. **Im Vergleich zu vor der OP, wie würden Sie Ihren jetzigen Gesundheitszustand beschreiben?**

**☐ Viel besser ☐ Etwas besser ☐ Unverändert zu vor der OP**

**☐ Etwas schlechter ☐ Viel schlechter**

1. **Inwieweit trifft jede der folgenden Aussagen auf Sie zu?**
   1. **Ich scheine etwas leichter als andere krank zu werden.**

**Trifft ganz zu  Trifft weitgehend zu  Weiss nicht
 Trifft weitgehend nicht zu  Trifft überhaupt nicht zu**

- 1. **Ich bin genauso gesund wie alle anderen.**

**Trifft ganz zu  Trifft weitgehend zu  Weiss nicht
 Trifft weitgehend nicht zu  Trifft überhaupt nicht zu**

- 1. **Ich erwarte, dass meine Gesundheit nachlässt.**

**Trifft ganz zu  Trifft weitgehend zu  Weiss nicht
 Trifft weitgehend nicht zu  Trifft überhaupt nicht zu**

- 1. **Ich erfreue mich ausgezeichneter Gesundheit.**

**Trifft ganz zu  Trifft weitgehend zu  Weiss nicht
 Trifft weitgehend nicht zu  Trifft überhaupt nicht zu**

**Sektion kognitive Funktionen:**

1. **Wie bewerten Sie Ihr Gedächtnis/Ihre Merkfähigkeit im Allgemeinen:**

| **Vor der OP** | **Gut** | **Schlecht** |  | **weiss nicht** |
| --- | --- | --- | --- | --- |
| **Nach der Operation** | **Besser als vor OP** | **Schlechter als vor OP** | **Unverändert zu vor der OP** | **weiss nicht** |

**Zum jetzigen Zeitpunkt auf einer Skala von 1 bis 10 (1 als Schlechtestes 10 als Bestes)?**

| **1** | **2** | **3** | **4** | **5** | **6** | **7** | **8** | **9** | **10** |
| --- | --- | --- | --- | --- | --- | --- | --- | --- | --- |

**Vor der OP, auf einer Skala von 1 bis 10 (1 als Schlechtestes 10 als Bestes)?**

| **1** | **2** | **3** | **4** | **5** | **6** | **7** | **8** | **9** | **10** |
| --- | --- | --- | --- | --- | --- | --- | --- | --- | --- |

1. **Wie bewerten Sie Ihr Kurzzeitgedächtnis:**

| **Vor der OP** | **Gut** | **Schlecht** |  | **weiss nicht** |
| --- | --- | --- | --- | --- |
| **Nach der Operation** | **Besser als vor OP** | **Schlechter als vor OP** | **Unverändert zu vor der OP** | **weiss nicht** |

**Zum jetzigen Zeitpunkt auf einer Skala von 1 bis 10 (1 als Schlechtestes 10 als Bestes)?**

| **1** | **2** | **3** | **4** | **5** | **6** | **7** | **8** | **9** | **10** |
| --- | --- | --- | --- | --- | --- | --- | --- | --- | --- |

**Vor der OP, auf einer Skala von 1 bis 10 (1 als Schlechtestes 10 als Bestes)?**

| **1** | **2** | **3** | **4** | **5** | **6** | **7** | **8** | **9** | **10** |
| --- | --- | --- | --- | --- | --- | --- | --- | --- | --- |

1. **Wie bewerten Sie Ihr Langzeitgedächtnis:**

| **Vor der OP** | **Gut** | **Schlecht** |  | **weiss nicht** |
| --- | --- | --- | --- | --- |
| **Nach der Operation** | **Besser als vor OP** | **Schlechter als vor OP** | **Unverändert zu vor der OP** | **weiss nicht** |

**Zum jetzigen Zeitpunkt auf einer Skala von 1 bis 10 (1 als Schlechtestes 10 als Bestes)?**

| **1** | **2** | **3** | **4** | **5** | **6** | **7** | **8** | **9** | **10** |
| --- | --- | --- | --- | --- | --- | --- | --- | --- | --- |

**Vor der OP, auf einer Skala von 1 bis 10 (1 als Schlechtestes 10 als Bestes)?**

| **1** | **2** | **3** | **4** | **5** | **6** | **7** | **8** | **9** | **10** |
| --- | --- | --- | --- | --- | --- | --- | --- | --- | --- |

1. **Gibt/gab es Situationen wo Ihr Gedächtnis besser/schlechter funktioniert?**

| **Vor der OP** | **Ja** | **Nein** | **weiss nicht** |
| --- | --- | --- | --- |
| Falls ja, in welchen  Situationen besser? |  | | |
| Falls ja, in welchen  Situationen schlechter? |  | | |
| **Zum jetzigen Zeitpunkt** | **Ja** | **Nein** | **weiss nicht** |
| Falls ja, in welchen  Situationen besser? |  | | |
| Falls ja, in welchen  Situationen schlechter? |  | | |

1. **Wie bewerten Sie Ihre rechnerischen Fähigkeiten:**

| **Vor der OP** | **Gut** | **Schlecht** |  | **weiss nicht** |
| --- | --- | --- | --- | --- |
| **Nach der Operation** | **Besser als vor OP** | **Schlechter als vor OP** | **Unverändert zu vor der OP** | **weiss nicht** |

**Zum jetzigen Zeitpunkt auf einer Skala von 1 bis 10 (1 als Schlechtestes 10 als Bestes)?**

| **1** | **2** | **3** | **4** | **5** | **6** | **7** | **8** | **9** | **10** |
| --- | --- | --- | --- | --- | --- | --- | --- | --- | --- |

**Vor der OP, auf einer Skala von 1 bis 10 (1 als Schlechtestes 10 als Bestes)?**

| **1** | **2** | **3** | **4** | **5** | **6** | **7** | **8** | **9** | **10** |
| --- | --- | --- | --- | --- | --- | --- | --- | --- | --- |

1. **Wie bewerten Sie Ihre Fähigkeit, Zusammenhänge zu erstellen/ Verknüpfungen zu machen?**

| **Vor der OP** | **Gut** | **Schlecht** |  | **weiss nicht** |
| --- | --- | --- | --- | --- |
| **Nach der Operation** | **Besser als vor OP** | **Schlechter als vor OP** | **Unverändert zu vor der OP** | **weiss nicht** |

**Zum jetzigen Zeitpunkt auf einer Skala von 1 bis 10 (1 als Schlechtestes 10 als Bestes)?**

| **1** | **2** | **3** | **4** | **5** | **6** | **7** | **8** | **9** | **10** |
| --- | --- | --- | --- | --- | --- | --- | --- | --- | --- |

**Vor der OP, auf einer Skala von 1 bis 10 (1 als Schlechtestes 10 als Bestes)?**

| **1** | **2** | **3** | **4** | **5** | **6** | **7** | **8** | **9** | **10** |
| --- | --- | --- | --- | --- | --- | --- | --- | --- | --- |

1. **Wie bewerten Sie Ihre Konzentrationsfähigkeit?**

| **Vor der OP** | **Gut** | **Schlecht** |  | **weiss nicht** |
| --- | --- | --- | --- | --- |
| **Nach der Operation** | **Besser als vor OP** | **Schlechter als vor OP** | **Unverändert zu vor der OP** | **weiss nicht** |

**Zum jetzigen Zeitpunkt auf einer Skala von 1 bis 10 (1 als Schlechtestes 10 als Bestes)?**

| **1** | **2** | **3** | **4** | **5** | **6** | **7** | **8** | **9** | **10** |
| --- | --- | --- | --- | --- | --- | --- | --- | --- | --- |

**Vor der OP, auf einer Skala von 1 bis 10 (1 als Schlechtestes 10 als Bestes)?**

| **1** | **2** | **3** | **4** | **5** | **6** | **7** | **8** | **9** | **10** |
| --- | --- | --- | --- | --- | --- | --- | --- | --- | --- |

1. **Wie bewerten Sie Ihre Fähigkeit alltägliche Aufgaben zu lösen?**

| **Vor der OP** | **Gut** | **Schlecht** |  | **weiss nicht** |
| --- | --- | --- | --- | --- |
| **Nach der Operation** | **Besser als vor OP** | **Schlechter als vor OP** | **Unverändert zu vor der OP** | **weiss nicht** |

**Zum jetzigen Zeitpunkt auf einer Skala von 1 bis 10 (1 als Schlechtestes 10 als Bestes)?**

| **1** | **2** | **3** | **4** | **5** | **6** | **7** | **8** | **9** | **10** |
| --- | --- | --- | --- | --- | --- | --- | --- | --- | --- |

**Ausgefüllt von:**

**Patient**

**Angehörige**

**Arzt**

**Vor der OP, auf einer Skala von 1 bis 10 (1 als Schlechtestes 10 als Bestes)?**

| **1** | **2** | **3** | **4** | **5** | **6** | **7** | **8** | **9** | **10** |
| --- | --- | --- | --- | --- | --- | --- | --- | --- | --- |

**Sektion Lebensqualität**

1. **Sind Sie in Ihrem Alltag selbstständig?**

**Ja  Nein**

- 1. **Falls nein, glauben Sie das dies mit der Operation zusammenhängt?**

**ja  nein  unbekannt/n.a.**

1. **Waren Sie vor der OP in Ihrem Alltag selbstständig?**

**Ja  Nein**

1. **Welche Fähigkeiten sind im Alltag nicht mehr vollständig selbstständig möglich?**

| **Einkaufen** | **Kochen** | **Essen & Trinken** | **Wäsche machen** |
| --- | --- | --- | --- |
| **Sich zuhause selbstständig fortzubewegen (Mobilität)** | **Medikamenten-einnahme** | **Geldgeschäfte** | **Körperpflege (sich baden oder anziehen)** |
| **Anstrengende Tätigkeiten (Sport treiben)** | **Haushaltsarbeiten (z.B. Reparaturen, Wohnung/Haus putzen)** | **Termine planen und einhalten** | **Mehr als eine Strassenkreuzung weit zu Fuss gehen** |

**War ein Teil von diesen Fähigkeiten vor der OP nicht möglich?**

**Ja  Nein**

**Wenn ja: Welche:**

1. **Auf einer Skala von 1 bis 10 (1= überhaupt nicht selbständig, 10=komplett selbständig), in welchem Ausmass können Sie Ihre alltäglichen physischen Aktivitäten (wie Gehen, Treppensteigen, Einkäufe tragen oder einen Stuhl verschieben) selbstständig durchführen?**

| **1** | **2** | **3** | **4** | **5** | **6** | **7** | **8** | **9** | **10** |
| --- | --- | --- | --- | --- | --- | --- | --- | --- | --- |

**Vor der OP, auf einer Skala von 1 bis 10 (1 als Schlechtestes 10 als Bestes)?**

| **1** | **2** | **3** | **4** | **5** | **6** | **7** | **8** | **9** | **10** |
| --- | --- | --- | --- | --- | --- | --- | --- | --- | --- |

1. **Auf einer Skala von 1 bis 10 (1 als Schlechtestes 10 als Bestes), wie gut können Sie Ihre gewohnten sozialen Aktivitäten und Rollen wahrnehmen?**

**(Dies beinhaltet Aktivitäten zu Hause, bei der Arbeit und Ihrem weiteren sozialen Umfeld. Auch sind hiermit Verantwortungen als Elternteil, Kind, Ehepartner/in, Angestellte, Freund/in etc. gemeint)**

| **1** | **2** | **3** | **4** | **5** | **6** | **7** | **8** | **9** | **10** |
| --- | --- | --- | --- | --- | --- | --- | --- | --- | --- |

**Vor der OP, auf einer Skala von 1 bis 10 (1 als Schlechtestes 10 als Bestes)?**

| **1** | **2** | **3** | **4** | **5** | **6** | **7** | **8** | **9** | **10** |
| --- | --- | --- | --- | --- | --- | --- | --- | --- | --- |

1. **Auf einer Skala von 1 bis 10 (1= überhaupt nicht, 10=sehr) wie sehr haben Ihre körperliche Gesundheit oder seelischen Probleme in den vergangenen 4 Wochen Ihre normalen Kontakte zu Familienangehörigen, Freunden, Nachbarn oder zum Bekanntenkreis beeinträchtigt?**

| **1** | **2** | **3** | **4** | **5** | **6** | **7** | **8** | **9** | **10** |
| --- | --- | --- | --- | --- | --- | --- | --- | --- | --- |

**Vor der OP, auf einer Skala von 1 bis 10 (1= überhaupt nicht, 10=sehr)?**

| **1** | **2** | **3** | **4** | **5** | **6** | **7** | **8** | **9** | **10** |
| --- | --- | --- | --- | --- | --- | --- | --- | --- | --- |

1. **Auf einer Skala von 1 bis 10 (1 als Schlechtestes 10 als Bestes), wie würden Sie Ihre Zufriedenheit mit Ihren sozialen Aktivitäten und Beziehungen einschätzen?**

| **1** | **2** | **3** | **4** | **5** | **6** | **7** | **8** | **9** | **10** |
| --- | --- | --- | --- | --- | --- | --- | --- | --- | --- |

**Vor der OP, auf einer Skala von 1 bis 10 (1 als Schlechtestes 10 als Bestes)?**

| **1** | **2** | **3** | **4** | **5** | **6** | **7** | **8** | **9** | **10** |
| --- | --- | --- | --- | --- | --- | --- | --- | --- | --- |

1. **Ist Ihre Lebensqualität nach der Operation:**

**Besser  Gleich  Schlechter  weiss nicht**

**Falls «besser» oder «schlechter», was hat sich verändert?**

**____________________________________________________________________________**

1. **Falls schlechter: Denken Sie, dass die Verschlechterung Ihrer Lebensqualität mit der Operation zusammenhängt?**

**Ja Nein  weiss nicht**

1. **Falls besser: Denken Sie, dass die Verbesserung Ihrer Lebensqualität mit der Operation zusammenhängt?**

**Ja Nein  weiss nicht**

1. **Auf einer Skala von 1 bis 10 (1 als Schlechtestes 10 als Bestes), wie würden Sie Ihre Lebensqualität heute einschätzen?**

| **1** | **2** | **3** | **4** | **5** | **6** | **7** | **8** | **9** | **10** |
| --- | --- | --- | --- | --- | --- | --- | --- | --- | --- |

**Vor der OP, auf einer Skala von 1 bis 10 (1 als Schlechtestes 10 als Bestes)?**

| **1** | **2** | **3** | **4** | **5** | **6** | **7** | **8** | **9** | **10** |
| --- | --- | --- | --- | --- | --- | --- | --- | --- | --- |

**Sektion Emotion**

Wie häufig kamen die folgenden emotionalen Zustände in den letzten 4 Wochen vor:

| **Voller Schwung** | **Immer** | **Meistens** | **Ziemlich oft** | **Manchmal** | **Selten** | **Nie** |
| --- | --- | --- | --- | --- | --- | --- |
| **Sehr nervös** | **Immer** | **Meistens** | **Ziemlich oft** | **Manchmal** | **Selten** | **Nie** |
| **Niedergeschlagen** | **Immer** | **Meistens** | **Ziemlich oft** | **Manchmal** | **Selten** | **Nie** |
| **Ruhig und gelassen** | **Immer** | **Meistens** | **Ziemlich oft** | **Manchmal** | **Selten** | **Nie** |
| **Voller Energie** | **Immer** | **Meistens** | **Ziemlich oft** | **Manchmal** | **Selten** | **Nie** |
| **Entmutigt und traurig** | **Immer** | **Meistens** | **Ziemlich oft** | **Manchmal** | **Selten** | **Nie** |
| **Erschöpft** | **Immer** | **Meistens** | **Ziemlich oft** | **Manchmal** | **Selten** | **Nie** |
| **Aggressiv** | **Immer** | **Meistens** | **Ziemlich oft** | **Manchmal** | **Selten** | **Nie** |
| **Glücklich** | **Immer** | **Meistens** | **Ziemlich oft** | **Manchmal** | **Selten** | **Nie** |
| **Müde** | **Immer** | **Meistens** | **Ziemlich oft** | **Manchmal** | **Selten** | **Nie** |

**Sektion Schmerzen**

1. **Wie stark waren Ihre Schmerzen:**

| **Vor der Operation** | **Keine** | **Sehr leicht** | **Leicht** | **Mässig** | **Stark** | **Sehr stark** |
| --- | --- | --- | --- | --- | --- | --- |
| **Nach der Operation** | **Keine** | **Sehr leicht** | **Leicht** | **Mässig** | **Stark** | **Sehr stark** |

**Im Vergleich zu vor der OP, sind ihre Schmerzen?**

**☐ Besser ☐ Unverändert ☐ Schlechter ☐ weiss nicht**

1. **Auf einer Skala von 1 bis 10 (1 = keine Schmerzen, 10 = sehr stark)?**

| **1** | **2** | **3** | **4** | **5** | **6** | **7** | **8** | **9** | **10** |
| --- | --- | --- | --- | --- | --- | --- | --- | --- | --- |

**Vor der OP, auf einer Skala von 1 bis 10 (1 = keine Schmerzen, 10 = sehr stark)?**

| **1** | **2** | **3** | **4** | **5** | **6** | **7** | **8** | **9** | **10** |
| --- | --- | --- | --- | --- | --- | --- | --- | --- | --- |

1. **Inwieweit haben die Schmerzen Sie in den vergangenen 4 Wochen bei der Ausübung Ihrer Alltagstätigkeiten zu Hause und im Beruf behindert?**

**Überhaupt nicht  Ein bisschen  Mäßig  Ziemlich
 Sehr**

**Sektion Schlaf**

Wie würden Sie ihre Schlafqualität einstufen:

| **Vor der OP** | **Gut** | **Schlecht** |  | **weiss nicht** |
| --- | --- | --- | --- | --- |
| **Nach der Operation** | **Besser als vor OP** | **Schlechter als vor OP** | **Unverändert zu vor der OP** | **weiss nicht** |

**Auf einer Skala von 1 bis 10 (1 als Schlechtestes 10 als Bestes)?**

| **1** | **2** | **3** | **4** | **5** | **6** | **7** | **8** | **9** | **10** |
| --- | --- | --- | --- | --- | --- | --- | --- | --- | --- |

**Vor der OP, auf einer Skala von 1 bis 10 (1 als Schlechtestes 10 als Bestes)?**

| **1** | **2** | **3** | **4** | **5** | **6** | **7** | **8** | **9** | **10** |
| --- | --- | --- | --- | --- | --- | --- | --- | --- | --- |

**Fazit**

**Würden Sie diese Operation weiterempfehlen?**

**Ja Nein  weiss nicht**

**NAAS Patient Satisfaction Index Score**

| **Die Behandlung hat meine Erwartungen erfüllt** | **1** |
| --- | --- |
| **Ich habe mich nicht so sehr verbessert, wie ich gehofft hatte, aber ich würde mich der gleichen Behandlung unterziehen, um das gleiche Ergebnis zu erzielen** | **2** |
| **Ich habe mich nicht so sehr verbessert, wie ich gehofft hatte, und ich würde mich nicht der gleichen Behandlung unterziehen, um das gleiche Ergebnis zu erzielen** | **3** |
| **Mir geht es genauso gut oder schlechter als vor der Behandlung** | **4** |

**Andere Bemerkungen:**

**___________________________________________________________________________**

Unterschrift und Datum Interviewer: _______________________________________
